# A Device-Independent Efficient Actigraphy Signal-Encoding System for Applications in Monitoring Daily Human Activities and Health

**DOI:** 10.3390/s18092966

**Published:** 2018-09-06

**Authors:** Yashodhan Athavale, Sridhar Krishnan

**Affiliations:** Department of Electrical, Computer and Biomedical Engineering, Ryerson University, Toronto, ON M5B 2K3, Canada; krishnan@ryerson.ca

**Keywords:** actigraphy, encoding, data compression, denoising, edge computing, signal processing, wearables, activity monitoring, machine learning

## Abstract

Actigraphs for personalized health and fitness monitoring is a trending niche market and fit aptly in the Internet of Medical Things (IoMT) paradigm. Conventionally, actigraphy is acquired and digitized using standard low pass filtering and quantization techniques. High sampling frequencies and quantization resolution of various actigraphs can lead to memory leakage and unwanted battery usage. Our systematic investigation on different types of actigraphy signals yields that lower levels of quantization are sufficient for acquiring and storing vital movement information while ensuring an increase in SNR, higher space savings, and in faster time. The objective of this study is to propose a low-level signal encoding method which could improve data acquisition and storage in actigraphs, as well as enhance signal clarity for pattern classification. To further verify this study, we have used a machine learning approach which suggests that signal encoding also improves pattern recognition accuracy. Our experiments indicate that signal encoding at the source results in an increase in SNR (signal-to-noise ratio) by at least 50–90%, coupled with a bit rate reduction by 50–80%, and an overall space savings in the range of 68–92%, depending on the type of actigraph and application used in our study. Consistent improvements by lowering the quantization factor also indicates that a 3-bit encoding of actigraphy data retains most prominent movement information, and also results in an increase of the pattern recognition accuracy by at least 10%.

## 1. Introduction

**Figure 1:**
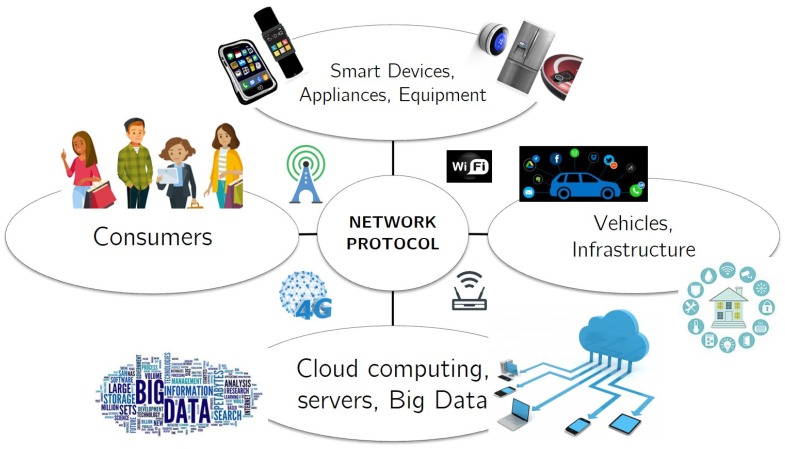
IoT Environment [4].

The advent of smart devices and rapidly evolving communication technologies, has enabled the formation of the Internet of Things (IoT) environment. The IoT paradigm intends to connect and exchange information and user data between devices, physical environment and the individual. This translates into a smart, connected and interactive environment for an individual, thereby improving the quality of life. The devices could be computers, phones, wearables, home appliances, infrastructure and vehicles [1,2,3]. Therefore, any device which operates even with an ON/OFF switch can be integrated into an IoT environment. The IoT environment also allows for connecting devices with limited memory, power and CPU. Figure 1 shows how different components and users are interconnected in an IoT paradigm [1,4].

**Figure 2:**
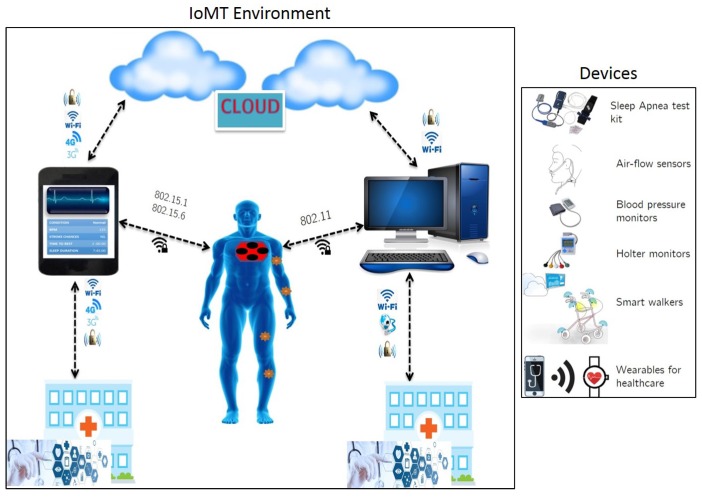
Connected Healthcare in an IoMT paradigm [5].

Advancements in sensor design have also enabled the rapid evolution of smart devices for personalized applications which include communication, health and fitness monitoring, virtual environments, autonomous transportation and smart homes. Considering the aspect of connected healthcare, the development of telehealth systems has resulted in coining of the term IoMT (Internet of Medical Things), which is a subset of IoT. The IoMT environment focuses on delivering clinical services to an individual via connected devices such as smart phones, wearables and infrastructure (see Figure 2). These services include [5]:

- Remote health monitoring via telecommunication network.
- Use of mobile health monitoring equipment and applications.
- Doctor-patient consultation via interactive technology.
- Continuous monitoring using smart devices for elderly and critical care individuals.

Our study is based on the use of wearables for home-based health monitoring in an IoMT environment. Wearables are devices embedded with accelerometers, gryoscopes, light and pressure sensors, for capturing and analyzing streaming physiological data from an individual during daily activity. Unlike smart phones or tablets, these devices can be comfortably worn on different body regions throughout the day, and can be used for various applications such as fitness monitoring, behavior tracking and vital signs analysis for critical disorders such as stroke, falls or seizures [6].

From our prior survey [6], we found that many currently available wearables such as Apple Watch^TM^ and FitBit^TM^ have embedded sensors for collecting and analyzing basic human activity parameters such as step counts, pulse rate, temperature and sleep times for fitness awareness. We also investigated into their respective SDKs (software development kits), which described how physiological data is collected, analyzed and shared with service providers for decision generation. In recent times, many clinical studies have been conducted to explore the validity of using wearables for physiological data analysis for disease or disorder detection. For example, accelerometer-based wearables have been used to study daily activity monitoring in individuals suffering from neuromuscular disorders, and validate their outputs with clinical standards [7].

As per a survey [8], considering that only about 90 out of 600 currently available wearables are being used for medical applications, we can see a clear potential for their usage in long-term, home-based health monitoring applications. Even though these numbers present a promising future for wearable-based health monitoring solutions, our review indicates that there still exist some crucial hurdles before implementing health monitoring devices and applications in real-time [6]. These include:

- Focusing on developing physiological signal analysis algorithms which promote edge computing approaches [4,5,6,9]. That is, the data acquisition, compression and analysis must be done at the device level without having the need to transmit long, streaming data to cloud services. This would lead to optimization of cloud resources by minimizing usage for data storage and analysis. The idea of edge computing is to help in optimizing on-device memory and power usage, thereby increasing operating efficiency and throughput [5,9].
- In addition to this, there is also a need for data acquisition standardization with respect to data formats and communication protocols [10,11].
- Ensuring seamless Internet connectivity across users, devices, infrastructure and services.
- Developing safe, non-invasive and comfortable wearables embedded with sensors for collecting and processing physiological data in a remote setting.

Meeting these challenges, could not only establish a set of standards with respect to device manufacturing and developing new communication protocols, but would also promote the development of novel data acquisition and storage algorithms in wearables. Since the most common sensor currently used in wearables is the accelerometer [6,8], we focus our study on activity monitoring applications. Note that wearables embedded exclusively with accelerometers are termed as actigraphs [12]. In the following section, we will discuss actigraphy applications, data acquisition and signal analysis.

## 2. Actigraphy

Actigraphs measure human body displacement in single or tri-axial directions, and have been used extensively in calculating gross motor activity for different applications. They are miniature devices which record and store motion data, which could then be further used for performing offline analysis. Actigraphs have been used by researchers in numerous clinical and consumer studies such as fitness monitoring, calorie consumption, sleep/wake activity analysis and for rehabilitation therapies in disabled individuals. To cite a few examples, actigraphy studies have been conducted in the following domains:

- Home-based sleep staging [13,14,15].
- Analyzing movements in individuals suffering from Parkinson’s and Alzheimer’s disease [16,17,18].
- Monitoring home activity of military personnel experiencing post-traumatic stress disorder (PTSD) [19].
- Routine of children diagnosed with autistic spectral disorder and ADHD (attention deficit hyperactivity disorder) [20,21].
- Estimating the severity of sleep related movement-disorders such as periodic limb movements (PLMs) [7,22,23].
- Therapeutic rehabilitation of joint disabilities in war veterans [24,25].
- Demographic studies for identifying differences in sleep patterns with respect to age, gender, ethnicity and sleep disorder prevalence [26].

**Figure 3:**
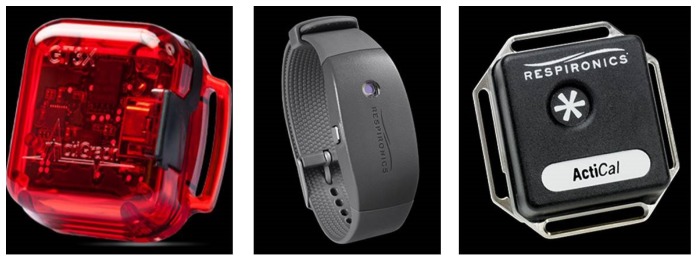
Example of actigraphs.

A variety of actigraphs are currently available in the market (see Figure 3), and they are usually worn on wrist, waist or lower ankles for capturing human motor activity [27]. Typically, an actigraph is able to capture motion data with a sampling frequency in the range of 16–3200 Hz, coupled with an A-to-D quantization of 6–16 bits per sample, depending on the manufacturer [7,12,27,28] .

The reader must note that, due to device property variability from one manufacturer to another, data analysis of the same activity captured from two different actigraphs, might yield different results. This infers that actigraphy analysis algorithms must be designed to be device-independent and customizable as per application [6,29]. Typically, an actigraph consists of the following components [12,30]:

- Piezoelectric accelerometer for capturing motion/vibrations.
- Signal amplifier coupled with an A-to-D converter.
- low-pass filter to remove external vibrations.
- Flash-memory to store sampled and filtered amplitudes.
- Capacitive and rechargeable battery.
- A micro-USB^TM^, serial or low power wireless interface to transfer data to a local computer.

**Figure 4:**
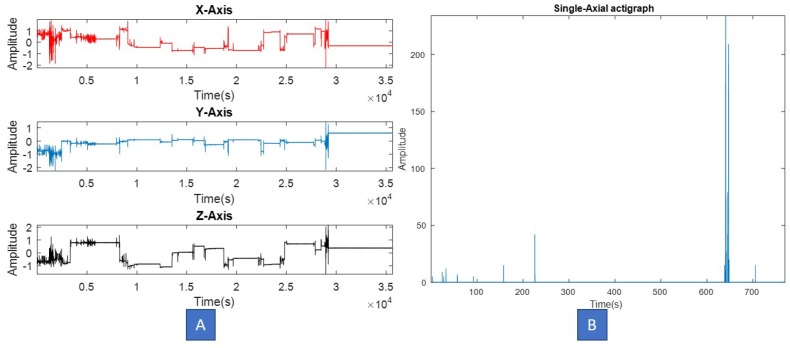
(**A**) Tri-axial, (**B**) Single axial actigraphy signal, captured from two different devices.

The actigraph maintains a record of zero-crossings and minimal thresholds, and uses them to generate raw signal values from the motion. Most of the currently available actigraphy devices are able to record and store 24 h motion data for up to a week. Depending on the choice and application domain, actigraphs could be single axial or tri-axial. Note that, usually tri-axial devices are comparatively more sensitive than single axial ones, and may capture motion in scenarios which require real-time data analysis. Figure 4 illustrates single and tri-axial actigraphy signals captured from two different actigraphs.

In case of tri-axial actigraphy data, our review of prior studies indicates that one must perform vector compounding of individual axial data before analysis, in order to simplify computations, and most importantly ensure that vibration information from all three directions is captured [14,31,32]. For example, given a tri-axial signal S=<x,y,z>, its vector magnitude would be computed as,

(1) $$V=\sqrt{x^{2}+y^{2}+z^{2}}$$

In order to analyze an actigraphy signal, we must first run certain signal property tests to determine appropriate processing tools and techniques [29]. Following Table 1 highlights various tests and our observations on actigraphy data, computed in MATLAB^TM^.

Before an actigraphy signal is analyzed to detect specific movements or patterns, it must be pre-processed in order to remove noise and artifacts. Conventionally, actigraphy signals undergo the following operations before analysis:

(1) A-to-D conversion in order to assign discrete amplitudes to specific movements [29].
(2) As per our literature review, human activity is usually captured in the 0.3 to 6 Hz frequency range, and high frequency noise is captured around the sampling frequency. In order to remove the noise, a simple low-pass filter (Butterworth) is employed to capture movement data [12,14,31,32].
(3) Additional band-pass filters could be implemented in order to remove low frequency artifacts and noise.
(4) Depending on application, the actigraphy signal is annotated using time-stamps. For example, in many sleep studies, actigraphy data was clipped between “Lights-off” and “Lights-on” time periods, in order to ensure alignment with other clinical signals recorded in simultaneous PSG [7].

Although most actigraphs are designed for long-term recordings, there are certain shortcomings in their data acquisition and storage methods, which need to be met in order to optimize their usage and implementation as standalone devices, or in smart wearables. These limitations could be:

(1) Actigraphs that sample data at higher frequencies (typically 100 Hz and above) along with a high quantization rate (typically 12–16 bits per sample), often lead to memory leakage and underutilization of battery life during recording.
(2) Manufacturer-based variability in sampling and quantization. This limits algorithms from being designed as device-independent tools [27,37]. Some actigraphs tend to sample movement data too infrequently, thus leading to information loss in the output raw signal.
(3) Many prior studies have been conducted on short-duration actigraphy datasets and did not require extensive memory and computational resources for analysis [14,22]. Translating these studies into long-term activity monitoring solutions is not feasible unless the actigraphy data is subjected to significant compression and segmentation at the source.
(4) Increased use of computational resources (local or cloud) during offline processing of long-term recordings. Conventionally, actigraphy data is captured and entirely transferred to a local computer or cloud for analysis. Our review indicates that in most studies, no prior data processing is done at the source to retain only meaningful information and discard redundant values.

As stated in previous section, signal acquisition methods which promote an edge computing approach could overcome the afore-mentioned challenges in long-duration actigraphy data analysis and optimize device usage [5,6]. In the following section, we propose one such technique to pre-processing actigraphy data by performing data compression and denoising at the source. It should be noted that the proposed solution in this study is not an edge computing technique in itself, but rather focuses on optimizing data acquisition and storage which would then promote edge computing on the hardware.

### Proposed Approach

In our review of actigraphy signals captured from different studies and applications, we found that employing a lower level of quantization to actigraphy data at the source, addresses a significant number of afore mentioned challenges. In this study, we propose a low-level encoding scheme which would improve actigraphy analysis in the following ways:

(1) Data compression at the source. The proposed encoding method intends to reduce the output actigraphy file size, thus enabling faster transfer and read time on a local computer.
(2) Signal normalization and denoising, which removes redundant and minute vibrations captured from highly sensitive accelerometers.
(3) SNR (signal-to-noise ratio) increase and enhancement of meaningful movement amplitudes in the signal.
(4) The proposed scheme also ensures operation across different types of actigraphs, thus promoting device-independency of this algorithm.

**Figure 5:**
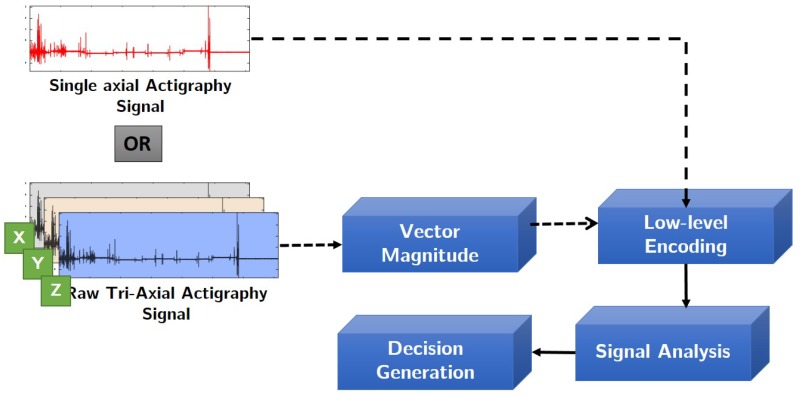
Flowchart of the proposed methodology.

The reader must note that data compression might result an increase in energy consumption and latency at the source. But the proposed solution intends to reduce memory usage and optimize overall battery usage, which would balance-off these shortcomings. Figure 5 illustrates the methodology implemented in this study.

In order to conduct a systematic investigation, we have conducted experiments on actigraphy data acquired from the following applications:

(1) Long-duration tri-axial actigraphy signals captured simultaneously with polysomnography in sleep studies [28].
(2) Activities of Daily Life (ADL) dataset obtained from Dua et al. [38].
(3) Vibroarthrographic signals captured from knee joints for osteoarthritis severity assessment [39].

The reader must note that in case of long-duration sleep actigraphy signals, the proposed encoding scheme’s results have already been published in [28] by Athavale et al., and hence we’ve shown the same results in this paper, to augment our experiments with daily activity [38] and vibroarthrography datasets [39].

For the reader’s reference, this paper has been further organized as follows: In Section 3.1 we will briefly explain the datasets used in our experiments, along with actigraph and signal properties used in each study. Next, in Section 3.2 we explain the proposed signal encoding scheme. Following this, we then proceed to check the validity of the proposed encoding scheme by performing simple machine learning and pattern classification of encoded signals, and comparing its results with those of raw actigraphy signals from each dataset, in Section 3.3 . In the next Section 4.1 and Section 4.2 we present our experimental results from signal encoding and its validation. We finally conclude this paper with some critical discussions in Section 5.

## 3. Materials and Methods

### 3.1. Data Acquisition

In the proposed study, we have conducted experiments on three datasets:

- Long-duration, tri-axial,bi-lateral ankle actigraphy signals [28]
- Short-duration, tri-axial, wrist-actigraphy signals [38], and
- Short-duration, single-axial, vibroarthrographic actigraphy signals [39]

Following Table 2 highlights describes the datasets used in our study:

In the next section, we will describe the proposed signal-encoding scheme applied to all the signals in the datasets described in Table 2.

### 3.2. Proposed Encoding Scheme

The proposed signal encoding scheme is then applied to afore mentioned actigraphy datasets as described in the following steps:

(1) The raw actigraphy signal is first normalized with respect to “g” factor using the device specifications. This operation removes signal components which have been amplified or caused due to earth’s gravitational effect on the accelerometer sensor [31]. In this study, depending on the application and device used, one of the following normalization step has been applied. Given a raw actigraphy signal $S_{r}=<x_{r},y_{r},z_{r}>$, its corresponding normalized version can be computed as follows:
  - For sleep, the normalized signal would be [28]
    (2) $$S=\frac{S_{r}}{2048\;counts/g}$$
  - For ADL, the normalized signal would be [38]
    (3) $$S=-1.5g+\frac{S_{r}}{63}\times 3g$$
  - For VAG, the signal is normalized as [39],
    (4) $$S=\frac{\left(max_{S_{r_{i}}}\left(S_{r}\right)-S\right)}{\left(max_{S_{r_{i}}}\left(S_{r}\right)-min_{S_{r_{i}}}\left(S_{r}\right)\right)}$$
    Note that in case of Eqns.2 and 3, $g=9.8\;m/s^{2}$.
  Note that the normalization operation is applied to each axis of the actigraphy signal.
(2) Next, depending on the signal type we perform vector compounding as shown in Equation (1). This operation is done only for tri-axial actigraphy data, and in case of single axial signals, we skip to normalization as shown in Equations (2)–(4).
(3) Assuming that *b* is the number of encoding bits, and $Q_{f}=\frac{2^{b}-1}{2}$ is the quantization factor, we encode the signal *S* using the floor operation,
  (5) $$S_{e}=\left\lfloor \left(S\times Q_{f}+Q_{f}\right)\right\rfloor$$
  The floor operation in Equation (5) digitally approximates each value generated from $\left(S\times Q_{f}+Q_{f}\right)$ to the greatest integer less than or equal to it. For example, a value of 3.4 would be mapped to 3. Note that in this study, we have experimented with different levels of encoding depending on the dataset. From our experiments, we have observed that a 3-bit encoding provides highest signal clarity.
(4) The SNR of the encoded actigraphy signal is then calculated as,
  (6) $${SNR}_{S_{e}}=20\log\left(\frac{{RMS}_{S}}{{RMS}_{Q_{e}}}\right)\;dB$$
  where, ${RMS}_{S}$ and ${RMS}_{Q_{e}}$ are the root mean square values of the input normalized signal and the quantization error respectively. The quantization error can be computed as $Q_{e}=\left(S-S_{e}\right)$.

The encoding scheme proposed in this section aims to perform on-the-fly denoising, SNR enhancement and compression of actigraphy data at the source. Our experimental results with different levels of encoding have been highlighted in Section 4.1. In the next section, we describe a validation process using a machine learning approach.

### 3.3. Validation Using Machine Learning

In order to ensure that no vital information is lost in the encoding process, we perform a machine learning validation in our study. This is done because unlike physiological data with characteristic patterns such as ECG, actigraphy signals do not show any specific structure or morphology, and hence obtaining a ground truth from experts proves to be trivial [29]. For example, in prior studies pertaining to actigraphy validation with PSG (polysomnography), clinical feedback was given only on PSG readings, and the actigraphy data was used only for comparing certain statistical parameters [7,23,40,41].

As shown in Table 1, the actigraphy data looks transient in nature, and requires ground truth information such as activity labels for further analysis. In order to validate the encoding scheme, we perform a simple feature extraction and pattern classification of raw and encoded actigraphy signals from each dataset used in this study, using the following steps:

(1) For each dataset, we create two distinct groups, namely:
  - Group 1: Raw actigraphy signals, and;
  - Group 2: Encoded actigraphy signals
(2) From each signal in Groups 1 and 2, we extract 13 time, frequency [7] and signal-specific features, defined in Table 3 as shown. For the reader’s reference, in this research study we propose two new signal specific features, namely—rapid change factor and spiky index. The remaining 11 features have been used in prior works pertaining to actigraphy and other physiological signal analysis applications [29].
(3) Next, depending on the dataset and its corresponding application, we apply pre-defined labels to Group 1 and 2 feature sets as follows:
  - **Sleep Data:** As the application is focused on distinguishing between mild and severe PLM (periodic limb movement) index, using the pre-defined labels in Athavale et al. [7,28], we divide the feature set into “Mild” and “Severe”.
  - **ADL Data:** Since this dataset contains signals of 14 multiple activities, we divide the feature set based on 14 labels [38].
  - **VAG Data:** As per Krishnan et al., the feature set has been divided into “Normal” and “Abnormal” depending on the severity of knee-joint degeneration [39].
(4) Finally, using a 70–30 ratio of training and testing feature data, we use an LDA (linear discriminant analysis) tool to classify actigraphy feature data within Groups 1 and 2 of each dataset. Further to this, we also cross-validate our results with a support vector machine (SVM).

It should be noted that in this study, machine learning of actigraphy data is not the main objective but has been used to validate the effect of signal encoding at source. Hence, the choice of using a LDA classifier has been done only to observe the linear classification performance on the encoded data. The results from this machine learning based validation for each dataset have been presented in Section 4.2.

## 4. Results

### 4.1. Signal-Encoding Results

**Figure 6:**
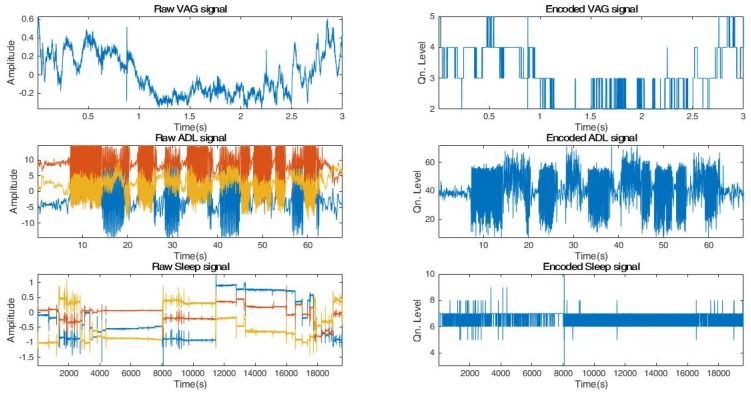
Sample Raw and Encoded signals from each dataset.

As evident from Equation (5), the encoding floor operation digitally approximates an actigraphy signal *S* by performing a non-linear mapping of each sample $S_{i}$ to an integer less than or equal to $S_{i}$ after multiplication with the quantization factor. Figure 6 illustrates a sample actigraphy signal from each dataset and its corresponding encoded version.

Additionally, we also perform a parameter-wise comparison, and observe that signal encoding not only inherently denoises and enhances SNR, but also performs significant data compression at the source. Following Table 4 highlights these results for a sample actigraphy signal obtained from each dataset.

**Figure 7:**
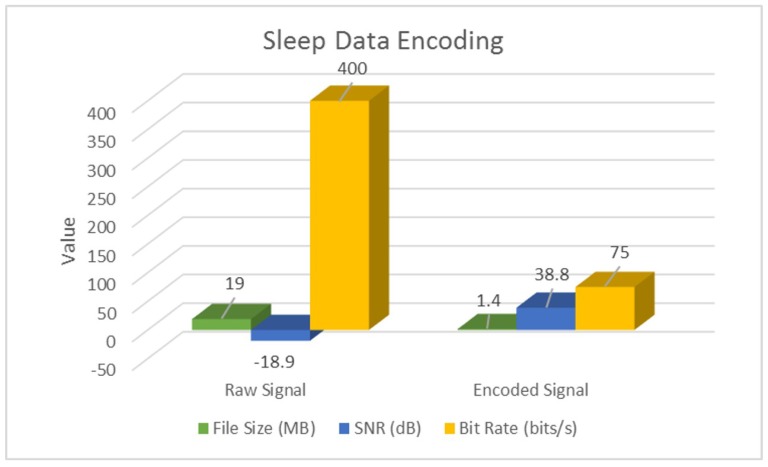
Encoding Sleep actigraphy signals.

**Figure 8:**
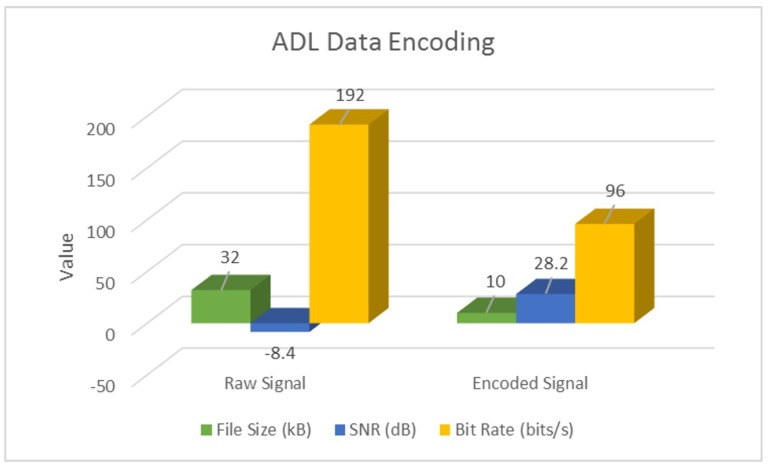
Encoding ADL actigraphy signals.

**Figure 9:**
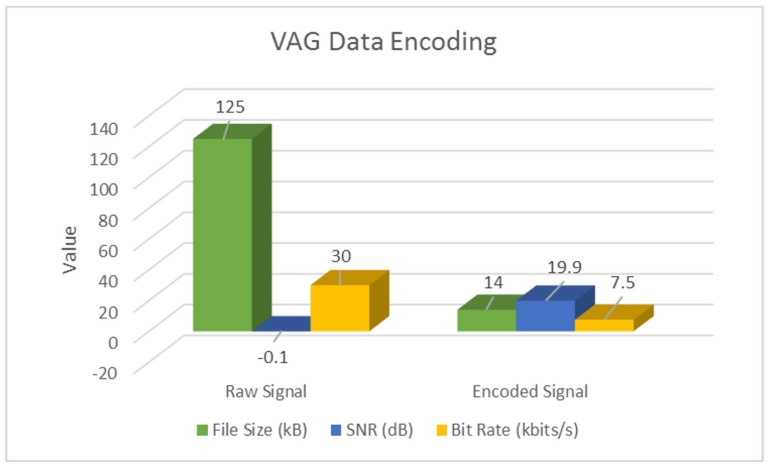
Encoding VAG actigraphy signals.

These results have also been illustrated in following Figure 7, Figure 8 and Figure 9.

As evident from Table 4 and Figure 7, Figure 8 and Figure 9, signal encoding not only enhances actigraphy data by retaining vital movement information and discarding redundant values, but also helps in signal compression at the source. Further to this, in Section 4.2, we highlight the machine learning validation results in order to show the encoding procedure’s efficiency in improving actigraphy signal recognition.

### 4.2. Encoding Validation Results

As described in Section 3.3, we performed a machine learning based validation of the proposed encoding scheme, and find that for each dataset, the classification rate within Group 2 (encoded) features is higher than that of Group 1 (raw) feature set. Table 5 highlights the classification results for LDA and SVM. In addition to computing the classification accuracies between raw and 3-bit encoded feature sets, we also calculate the F1-score metric for each data-set’s classification rate using the expression,

(7) $$F1=2\times \frac{Precision\times Recall}{Precision+Recall}$$

As evident from Table 5, the classification accuracies for ADL data [38] does not increase significantly even after encoding. We investigated this further and found that the classification rates varied drastically within the 14 classes of the ADL data due to lack of sufficient number of signals for certain activities. Nevertheless, we have still included the encoding results in this study, in order show the applicability of the proposed technique to any type of actigraphy.

**Figure 10:**
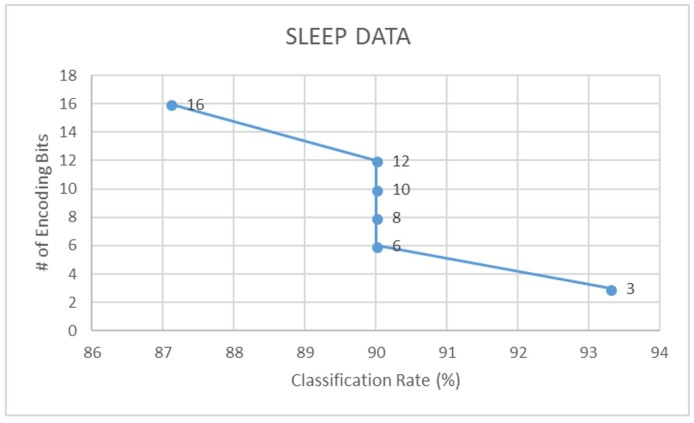
Classification rate vs. encoding - sleep data.

**Figure 11:**
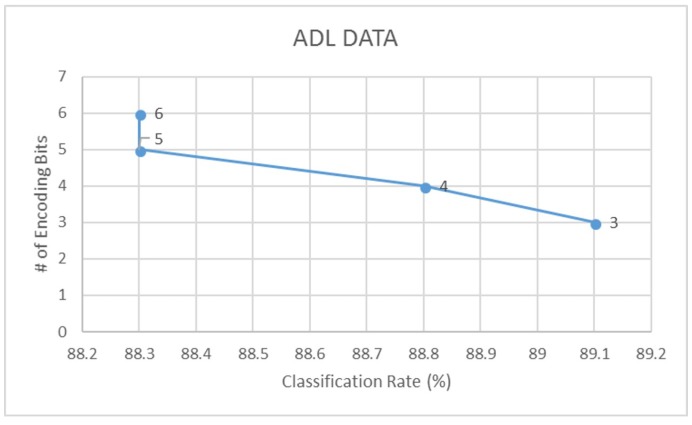
Classification rate vs. encoding - ADL data.

**Figure 12:**
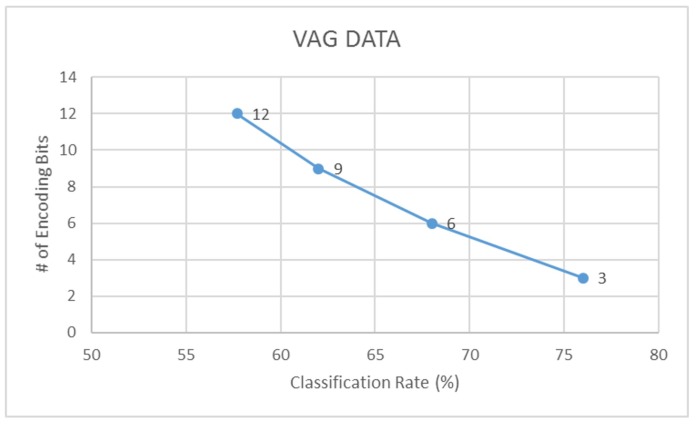
Classification rate vs. encoding - VAG data.

Further to this, we also compare the LDA classification accuracies of signals encoded using different bit-factors for each dataset. Through this, we find that a 3-bit encoding of actigraphy data ensures highest performance in data acquisition, storage and analysis. Following Figure 10, Figure 11 and Figure 12 illustrates this trend on how the classification rate for each dataset decreases with increase in bit resolution of the signal.

## 5. Discussions and Future Works

As evident from our investigation and experimental results, employing a very low-factor signal quantization greatly improves the device’s data handling capacity by ensuring enhanced SNR, high compression ratio and removal of redundant movement information from the actigraphy signal. The 3-bit encoding proposed in this study, works best in compressing actigraphy data at the edge of an IoT-type setup. Considering the nature of actigraphy signals as highlighted in Table 1, the proposed encoding scheme addresses the transient, spiky information by retaining only significant movement amplitudes or true acceleration values. Movements which are very small are floored to zero in the encoding operation. Thus, redundant values and high frequency noise are removed in the encoded signal, which now contains only relevant movement information.

Although in this study we have used offline datasets, it must be noted that the objective of the proposed encoding scheme is to be applied at the recording source (i.e., on the device) in real-time. This supports an edge computing approach when coupled with activity-based adaptive segmentation techniques to extract regions of peak movements. The machine learning validation approach used in this study aptly supports the proposed encoding scheme as shown by the classification results in Table 5. Further to this, we observe that the 3-bit encoding provides the highest activity recognition rate. From our study on different actigraphy datasets, it should be noted that the proposed encoding algorithm is device-independent and signal-independent, and could easily be ported onto any accelerometer-based wearable.

Current trends in IoMT and related device developments highly promote the edge computing structure in smart devices, as it would significantly reduce cloud burden, and ensure data privacy and security at the consumer end. Home-based health monitoring using an IoMT framework is a burgeoning market and would help in significant reduction of patient-doctor visits and associated healthcare costs. One way to encourage this trend is to use wearables and sensors, embedded with edge computing friendly algorithms, such as the one proposed in this study. This would also promote the clinical validation and development of tools for long-term monitoring of vital physiological parameters in not just chronically ill or elderly patients, but for the betterment of all individuals [6,43].

As part of our future work, we would like to test the proposed algorithm’s efficiency on commercially available wearables such as FitBit^TM^, Apple Watch^TM^ as well as other generic actigraphs used in activity monitoring studies.

## Figures and Tables

**Table 1:** Actigraphy signal tests.

Property Test	Observations
Visual inspection	Spiky data with a lot of transient information randomly distributed. Motion events seem uncorrelated when separated by significant time period.
Stationarity—KPSS test [33]	Non-stationary signals
Linearity—Augmented Dickey–Fuller test [34]	Non-linear data
Gaussianity—KS test [35]	Non-Gaussian distribution in most cases, since human motion is random.
Sparsity test—Gini Index [36]	Sparse in short windows. In case of tri-axial data, vector compounding and additional quantization may be needed.

**Table 2:** Dataset Properties.

Application	Data-Type	No. of Signals	Length/Signal	Resolution	$f_{s}$
Sleep [28]	Tri-axial	50	6–8 h	16-bits/sample	25 Hz
ADL [38]	Tri-axial	274	5–60 s	6-bits/sample	32 Hz
VAG [39]	Single-axial	89	3–5 s	12-bits/sample	2 kHz

$f_{s}$ is the sampling frequency.

**Table 3:** Features and their description

Domain	Feature	Description
**Time**	RMS	Root mean square value of the signal
	Maxima	Maximum Peak value in the signal
	Peak-to-Peak	Difference between maximum and minimum peak
	Peak-to-RMS	Maximum peak to RMS ratio
	Peak-to-Avg.Power	Maximum peak to avg. power ratio
	SNDR	Signal to noise & distortion ratio
	Hjorth’s Parameters [42]	First order mobility, $M_{f}=\sqrt{\frac{\sigma_{f}}{\sigma_{x}}}$
		Second order mobility, $M_{s}=\sqrt{\frac{\sigma_{s}}{\sigma_{x}}}$
		Complexity, $C_{x}=\frac{M_{fs}}{M_{f}}$
**Frequency**	Median Frequency	Median normalized frequency of power spectrum
	Band power	Average signal power
**Signal-Specific**	Spiky Index	$SI=\frac{\#\;of\;Prominent\;Peaks\;or\;events}{Total\;Activity\;Time(s)}$
	Rapid Change Factor	$RCF=\frac{Step\;Size}{b\times T_{s}}$

**Table 4:** Parametric Encoding Results.

Signal Type	Parameter	Sleep	ADL	VAG
**Raw**	SNR (dB)	−18.9	−48.4	−0.1
	Bit Rate (bits/s)	400	192	$20\times 10^{3}$
**Encoded**	SNR (dB)	38.8	28.2	19.9
	Bit Rate (bits/s)	75	96	$6\times 10^{3}$
**Overall**	% Space Savings	92%	68%	88%

**Table 5:** Machine Learning Results along F1-Scores for each actigraphy dataset.

Data	Raw Features				Encoded Features			
	LDA		SVM		LDA		SVM	
	Accuracy (%)	F1-Score	Accuracy (%)	F1-Score	Accuracy (%)	F1-Score	Accuracy (%)	F1-Score
Sleep	87.1	0.78	83.3	0.71	93.3	0.90	93.3	0.91
ADL	88.3	0.82	82.8	0.73	89.1	0.85	84.9	0.76
VAG	57.7	0.45	65.4	0.59	76.0	0.70	84.6	0.81

## Abbreviations

The following abbreviations are used in this manuscript:

IoT	Internet of Things
IoMT	Internet of Medical Things
VAG	Vibroarthrography
ADL	Activities of Daily Life
SNR	signal-to-noise ratio
LDA	linear discriminant analysis
SVM	support vector machine
